# Trace Elements in Soy-Based Beverages: A Comprehensive Study of Total Content and In Vitro Bioaccessibility

**DOI:** 10.3390/ijerph20064986

**Published:** 2023-03-12

**Authors:** Raquel Fernanda Milani, Adriana Aparecida Mauri, Vitor Lacerda Sanches, Marcelo Antonio Morgano, Solange Cadore

**Affiliations:** 1 Institute of Food Technology (ITAL), P.O. Box 139, Campinas 13070-178, SP, Brazil; 2 Institute of Chemistry, University of Campinas, P.O. Box 6154, Campinas 13083-970, SP, Brazil

**Keywords:** soy-based beverages, trace elements, bioavailability, nutritional quality, food safety, ICP OES

## Abstract

Soy-based beverages are one of the most consumed plant-based beverages, which have been used as a substitute for dairy products. Soy is a source of several nutrients (vitamins, minerals, and phenolic compounds, etc.) and its consumption is usually associated with several benefits, such as the prevention of cardiovascular diseases, cancer, and osteoporosis. However, non-essential trace elements can be found in these beverages. Thus, a comprehensive study concerning trace elements Al, As, Cd, Co, Cr, Cu, Fe, Li, Mn, Ni, Pb, Sb, Se, Sn, Sr, and Zn in soy-based beverages was proposed. In vitro digestion allowed to simulate the gastrointestinal juice (bioaccessibility) and the Caco-2 cells culture model was applied for the bioavailability assay. Trace elements measures were performed by inductively coupled plasma optical emission spectrometry (ICP OES). Multivariate analysis classified soy-based beverages according to their soy source (isolate protein, hydrosoluble extract, and beans); Al, Cu, Fe, Mn, Sr, Se, and Zn bioaccessible fractions corresponded to approximately 40%-80% of their total content, and soy-based beverages were found to be a good Fe, Se, and Zn source. However, our results showed risk exposure assessment from daily consumption of one glass of soy-based beverage can contribute to 3.5% and 0.9% of Al Provisional Tolerable Weekly Intake (PTWI) for children and adults, respectively.

## 1. Introduction

Soy-based beverages are one of the most consumed plant-based beverages, a global trend in the food market which represent an alternative for dairy products [1]. Its composition does not contain cholesterol, gluten, and lactose, being adequate to restricted diets [2]. The main soy sources in these beverages are the beans, the hydrosoluble extract, and the isolate protein. The hydrosoluble extracts are obtained by maceration and hydration of the soybeans, including a peeling step, which attenuates the bitterness and the astringent taste. The isolate protein is an end product of soy processing, containing more than 90% of protein, in dry base [3].

Soy-based products are usually associated with the prevention of cardiac diseases, cancer, osteoporosis, and the reduction in the menopause symptoms [4]. Besides its nutrient composition, including minerals, vitamins, and antioxidant compounds, non-essential trace elements may be found in soy-based beverages. Environmental pollution, soil, irrigation water, and food industry equipment are known sources of these contaminants [5].

After ingestion, food components undergo a complex biotransformation process, and trace element contents in food usually do not represent the fraction available for absorption (bioaccessible) or the fraction that can be absorbed by the intestine for biological functions (bioavailable) [6,7]. In vitro digestion combined with an intestinal epithelial model, such as the Caco-2 cells culture, is one of the main preliminary assays employed for bioavailability [5,7].

In vitro digestion models were recently applied for elements evaluation in soy and soy-based beverages. Herrera-Agudelo et al. [8] studied cultivated transgenic and not-transgenic soybeans and reported Cu, Fe, Mn, and Zn bioaccessibility ranging from 26% to 107.7%. Theodoropoulos et al. [9] combined the in vitro digestion and the dialysis assay and found values ranging from ND to 27.5% and 7.6 to 22.2% for Fe and Zn, respectively. Similar values for Zn bioaccessibility were also reported by Sanches et al. [10] and by Silva et al. [1], corresponding to 18–21% and 6.15% of its total content, respectively.

In cereals, polysaccharides associated to proteins, phenolic compounds, and phytic acid (antinutritional compound) may modify the ability of the complexation of mineral and alimentary fibers, such as inulin [11]. The main inositol forms in soy-based beverages usually are myo-inositol hexakis (IP6) and pentakisphosphate inositol (IP5), ranging from 5.0 to 19.7 and 0.2 to 5.2 µmol/g, respectively [9], agreeing with the consensus that phytates have different complexation capacities. For example, myo-inositol tetrakisphosphate (IP4) appears to present no negative effects to mineral absorption [12]. Phytase addition in soy-based beverages was also reported in literature as an approach to increase mineral bioaccessibility, achieving values up to 37% and 67% for Fe and Zn bioaccessibility, respectively [9].

Although the relevance of studies regarding trace elements in plant-based beverages is clear, there is still a lack of data in the literature. To the best of our knowledge, no comprehensive data have been reported regarding the bioavailability of trace elements (Al, As, Cd, Co, Cr, Cu, Fe, Li, Mn, Ni, Pb, Sb, Se, Sn, Sr, and Zn) in soy-based beverages. This study aimed to assess the total content of these trace elements and to evaluate the effect of in vitro digestion in their bioavailability. An analysis was performed by ICP OES, and the estimation of risk related to trace elements, inorganic contaminants exposure, and the mineral dietary intake from soy-based beverage consumption was performed, considering adults and children.

## 2. Materials and Methods

### 2.1. Samples and Reagents

Soy-based beverages were acquired in markets from Campinas, SP (Brazil), equally distributed from the different soy sources (isolate protein, hydrosoluble extract and soybeans), totaling 18 samples (Table 1). Sampling considered two different batches from main manufactures, in three available flavors: apple, grape and orange. The beverage samples were immediately analyzed when their packaging was opened.

For trace elements analysis, reversed osmosis water (Gehaka, São Paulo, Brazil) and sub-boiled nitric acid (Berghof, Eningen, Germany) were employed. ICP standard solutions of Sb and Se (1000 mg/L, Fluka, Steinheim, Germany) and multielementar (1000 mg/L, Merck, Darmstadt, Germany) were used for analytical curves (at least 5 points): 1.0–500 µg/L for As, Cd, Co, Cr, Li, Ni, Pb, Sb, Se, Sr; 1.0–1000 μg/L for Cu, Sn, Zn; 2.5–5000 µg/L for Al, Fe, Mn.

For the bioavailability study, enzymes (pancreatin from porcine pancreas—3xUSP, pepsin from porcine gastric mucosa—480 U/mg, and bile extract from bovine and ovine) and Dulbecco’s Modified Eagle’s Medium high glucose (DMEM) were acquired from Sigma-Aldrich (Buchs, Switzerland); 37% hydrochloric acid and 30% hydrogen peroxide from Merck (Darmstadt, Germany); ammonium bicarbonate from Carlo Erba (Milano, Italy); inactive fetal bovine serum, sterile solutions (0.2 g/L EDTA and 2.5 g/L trypsin) from Cultilab (Campinas, Brazil); Dulbecco Phosphate Buffered Saline (D-PBS without Ca^2+^ and Mg^2+^) from Nutricell (Campinas, Brazil); DMEM supplementary solutions (100 mmol/L sodium pyruvate, 200 mmol/L L-glutamine, 1 mol/L HEPES, 100 UI/mL penicillin, 100 µI/mL streptomycin and non-essential amino acids—NEAA 100x) from Gibco (Grand Island, NE, USA).

For phytic acid analysis, sodium chloride and hydrochloric acid (Merck, Darmstadt, Germany) and Dowex^®^ G-55 chloride form resin (Sigma Aldrich, SL, USA) were employed. The analytical curve was prepared using 10,000 mg/L P standard solution (Specsol, São Paulo, Brazil) in 0.01–10.0 mg/L range (7 points) in 0.7 mol/L NaCl (*w*/*v*)/5% (*v*/*v*) HNO_3_ medium.

### 2.2. Analytical Methods

Analyses were carried out by ICP OES (model 5100 VDV, Agilent Technologies, Tokyo, Japan). The optimized conditions are briefly described in Table 2 [13].

For trace element determination, soy-based beverages were digested by a microwave digestion system (Start D, Milestone, Sorisole, Italy). Briefly, 2 mL of sample and 10 mL of HNO_3_ + H_2_O_2_ were added to the digestion vessel (PTFE). After the decomposition, the solution was diluted to 20 mL (volumetric flask) using reversed osmosis water. To analyze the bioaccessible extracts, acid digestion was performed employing an aliquot of 4 mL [13].

#### 2.2.1. Bioaccessibility Assay

For the bioaccessibility assay, an in vitro static digestion method [5,14,15] was employed, simulating the gastrointestinal phase of human digestion. The pH was adjusted using a selective electrode (model Starter 3100, Ohaus, Barueri, Brazil), and a water bath under agitation (Tecnal, Piracicaba, Brazil) was employed.

Briefly, 10 mL of sample were transferred to a conical flask and the pH was adjusted to 2.0 (6 mol/L HCl). A solution of pepsin was added (0.01 g pepsin/5 mL sample) and the flask was incubated at 37 °C for 2 h. Then the pH was adjusted to 5.0 (20% NH_4_OH). An enzymatic solution (0.0025 g pancreatin +0.015 g bile extract/5 mL sample) was added, and the flask was incubated (2 h). The pH was verified and, if necessary, adjusted to 7.0 (20% NH_4_OH). The gastrointestinal extract was centrifuged at 3500 rpm for 30 min (Fanem, São Paulo, Brazil) and the supernatant was filtered (0.45 µm). The percentage of the bioaccessible concentration was calculated as a ratio between the concentrations of trace elements in bioaccessible fraction and their total content (soy-based beverage).

#### 2.2.2. Bioavailability Assay

For the bioavailability assay, a method described in our previous study was used [5]. Briefly, Caco-2 cell cultures (Rio de Janeiro Cell Bank, passage 49) were preserved in DMEM + supplementary solutions at 37 °C under 5% CO_2_ atmosphere (Sanyo, Moriguchi City, Japan) [7,15]. After reaching 70% confluence and minimum density of 5 × 10^4^ cells/cm^2^, Caco-2 cells were seeded in 24 mm 6 well Transwell^®^ plates (0.4 μm, Corning Inc., New York, NY, USA). A differentiation period was achieved after 21 days, and microbiologic contamination was evaluated daily using optical microscopy. For experiments, osmolality of the extract was verified (model K-7400, Knauer, Berlin, Germany) and, if necessary, adjusted to 310 ± 10 mOsm/kg using NaCl. To Transwell^®^ plates; 1.5 mL of the extracts, and 2.0 mL of a synthetic fluid (0.35 g/L NaHCO_3_, 0.7 g/L KCl, 1.0 g/L glucose and 8.0 g/L NaCl) were added to the apical and basolateral portions, respectively. After 2 h of incubation, the contents of these portions were separated for trace elements analysis.

#### 2.2.3. Phytic Acid Analysis

For the phytic acid assay, 0.5 mL soy-based sample and 9.5 mL of 2.4% HCl (*v*/*v*) were added to a graduated tube and kept in orbital agitation (Marconi, São Paulo, Brazil) for 1 h. The solution was filtered and percolated in a Dowex^®^ G-55 chloride form resin column. The retained content was eluted employing a 0.7 mol/L NaCl (*w*/*v*) solution and separated for further phosphorus analysis by ICP OES [16,17,18].

### 2.3. Quality Assessment

Method development and quality control were reported in our previous study [13]. All analyses were performed in triplicate, and blank experiments followed the procedure described in Section 2.2.1, Section 2.2.2 and Section 2.2.3. The analytical methods were evaluated according to the INMETRO [19]: limits of detection (LOD) = 0 + t(*n* − 1, 1 − α).s and limits of quantification (LOQ) = 10s, being “s” = standard deviation of blank experiments and t = 3.143 (at 99% confidence level); certified reference materials Corn Bran (RM 8433, NIST, Gaithersburg, MD, USA), Tea leaves (INCT-TL-1, Instytut Chemii i Techniki Jądrowej, Warszawa, Poland) and spiked experiments were also made for accuracy and precision (coefficient of variation, in percentage).

Results demonstrated conformity with the Brazilian [20], MERCOSUR [21], and European [22] thresholds: LOD and LOQ values were 0.3–12.0 µg/L and 1.1–38.2 µg/L, respectively, and AOAC [23] recovery values were 79–118%. Precision was below 10% for the majority of the elements studied.

### 2.4. Statistical Analysis

One-way analysis of variance (ANOVA) and Tukey’s test used XLSTAT software 2017.7.48873 (Addinsoft, Paris, France). For multivariate analysis, Pirouette software 3.11 (Infometrix, Woodinville, WA, USA) was used, displaying the auto escalated data in a matrix composed of 18 lines and 8 columns. According to the Mahalanobis distance, no outliers were found.

## 3. Results and Discussion

### 3.1. Trace Element Contents in Soy-Based Beverages

Trace element contents (mean and range) in soy-based beverages are described in Table 3. For trace element contents lower than the limit of quantification, results were expressed as “<” their LOQ.

From Table 3, inorganic contaminants such as arsenic, cadmium, lead, and tin were found within the thresholds established by Brazilian [20] and MERCOSUR [21] regulations: As (0.05 mg/kg), Cd (0.02 mg/kg), Pb (0.05 mg/kg), and Sn (150 mg/kg). Low levels were also found for Co, Cr, Li, Ni, and Sb in soy-based beverages, ranging from <LOQ to 61 µg/L and being similar, according to the ANOVA and Tukey’s test at 95% of the confidence level.

Nonetheless, for Al, Cu, Fe, Mn, Se, Sr, and Zn wide ranges were observed: levels varied up to 40x between minimum and maximum values, indicating possible influence of soy source used in these plant-based beverages. The highest values of Al and Se were found in beverages with isolated soy protein (1822 µg/L and 110 µg/L, respectively) whilst the highest levels of Cu, Fe, and Mn were found in beverages with soy hydrosoluble extract (268 µg/L, 11,385 µg/L, and 665 µg/L, respectively). Zinc mean levels were similar between isolate soy protein and soybean beverages, being 4205 and 4893 µg/L, respectively. For Sr, the results indicated a possible correlation with amino acids present in soy protein: similar levels were found in isolate soy and soybean beverages, ranging from 213 to 699 µg/L.

Although soy-based foodstuffs are widely consumed, there are few studies reported concerning trace elements composition. Concerning soy-based food, Barbosa et al. [24] studied soy extract, soy protein, soybean, and whole soy flour, and reported similar values for soy extract (8.6 and 11.5 mg/kg for Cu, 24.9 and 56 mg/kg for Fe, 17.8 and 21.9 mg/kg for Mn, 1.87 and 2.55 mg/kg for Ni, <2.90 and <2.90 mg/kg for Se, 3.47 and 5.34 mg/kg for Sr, 29.3 and 39.3 mg/kg for Zn, <0.007 and <0.007 mg/kg for As, <0.0059 and 0.028 mg/kg for Cd, 0.0229 and 0.14 mg/kg for Co, 0.69 and 3.4 mg/kg for Cr, <0.029 and 0.038 mg/kg) and even higher levels in soybean: Cu (7.0 and 11.0 mg/kg), Fe (46 and 124 mg/kg), Mn (17.2 and 35.2 mg/kg), Ni (2.5 and 4.1 mg/kg), Se (18 and 25 mg/kg), Sr (4.1 and 7.5 mg/kg), Zn (30.8 and 42.6 mg/kg), As (0.011 and 0.040 mg/kg), Cd (<0.006 and 0.019 mg/kg), Co (21.0 and 100 mg/kg), Cr (5.5 and 10 mg/kg), and Pb (<0.029 and 0.035 mg/kg).

In general, higher levels of trace elements than our study were reported by Llorent-Martínez et al. [25] in soy-based food acquired in Spain: Al (900–3000 µg/kg), As (2–4 µg/kg), Cd (1.5–5 µg/kg), Co (5–40 µg/kg), Cr (10–55 µg/kg), Cu (700–1300 µg/kg), Fe (4000–9400 µg/kg), Mn (1300–2400 µg/kg), Ni (100–500 µg/kg), Pb (2–8 µg/kg), Sb (<1.2–1.5 µg/kg), Sn (<1.5 µg/kg), and Zn (2100–3200 µg/kg). Boa Morte et al. [26] also reported high levels of Al (0.98–2.02 mg/L), Cu (0.412–0.907 mg/L), and Fe (3.06–5.92 mg/L) in soy formulations from Bahia (Brazil), and Andrés et al. [27] reported higher Zn levels in soy-based beverages containing fruit juice than in similar milk beverages.

**Figure 1:**
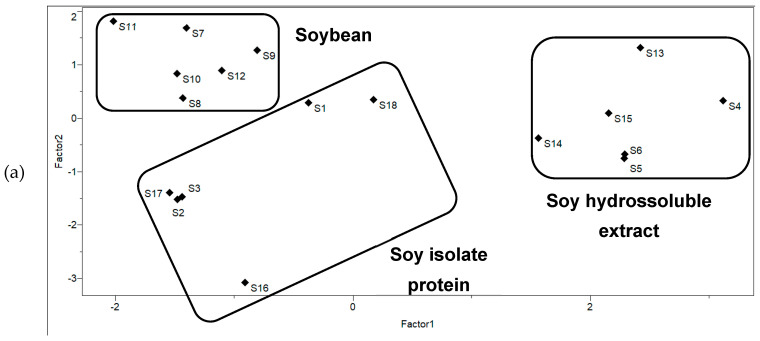

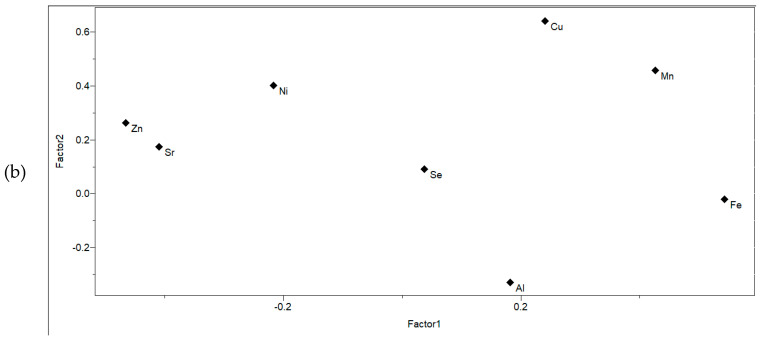
Principal component analysis for trace elements in soy-based beverages: (**a**) scores plot; (**b**) loading plot.

Considering the elements with quantifiable levels (Al, Cu, Fe, Mn, Ni, Se, Sr, and Zn), principal components analysis (PCA) was performed in order to verify similarities in soy-based beverage composition. In Figure 1, the scores and loadings plots were provided. The total variance (81.38%) was explained by principal components PC 1 (Factor 1 = 52.90%) and PC 2 (Factor 2 = 28.48%), being related to Fe (0.5426), Mn (0.4261), Zn (−0.4657), Sr (−0.4097), and Cu (0.6412), Mn (0.4575), Ni (0.4031), Al (−0.3290), respectively. Information in the parentheses (negative or positive) corresponds to the loadings values.

From Figure 1, three groups were classified:

- Group 1 (S7, S8, S9, S10, S11, and S12): Soybean beverages: associated with Sr, Ni and Zn (positive loadings in Factor 2);
- Group 2 (S4, S5, S6, S13, S14 and S15): Beverages with soy hydrosoluble extract: associated with Fe and Mn (positive loadings in Factor 1) and to Zn (negative loading in Factor 1);
- Group 3 (S1, S2, S3, S16, S17 and S18): Isolate soy protein beverages: associated mainly with Al and Zn (positive loadings in Factor 1 and 2).

### 3.2. Trace Elements Bioavailability from Soy-Based Beverages

**Figure 2:**
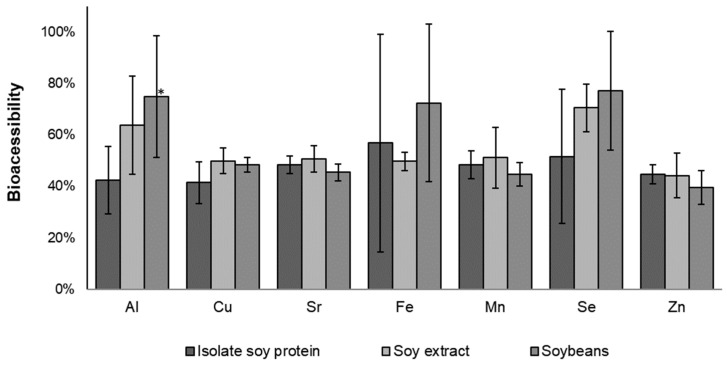
Trace elements bioaccessibility from soy-based beverages. * Mean values between bar with same pattern are significantly different at *p* < 0.05, according to Tukey’s test. Error bars represent standard deviation.

Bioaccessibility studies considered the trace elements with quantifiable levels (Al, Cu, Fe, Mn, Sr, Se, and Zn). The results are shown in Figure 2.

Overall, the bioaccessible fractions of Cu, Sr, Mn, and Zn in soy-based beverages corresponded to 39–51% of their total content, inferring a low influence of the soy source in these beverages. Zinc bioaccessible contents are in accordance with Sanches et al.’s [10] study, which found values between 18 and 21% of their total content.

Nevertheless, for Al and Se, a pattern of bioaccessibility was found in isolate soy protein < soy hydrosoluble extract < soybeans, indicating an interaction between these elements and the soy protein, which corresponds to 90% in isolate soy protein [3].

For Fe, one could notice large bioaccessibility ranges, and this finding is in accordance with the established interaction between trace elements and polyphenolic compounds in vegetables [28]. Organic acids were also reported as food components with ability to complexation metallic ions, being present in soy-based beverages with fruit juice addition: citric acid from orange juice, malic acid from apple juice, and tartaric acid from grape juice [27,29].

Bioavailability experiments were performed using Caco-2 cell cultures and the contents of the apical (retention) and basolateral (transport) layers were measured. The quantifiable elements in basolateral layer (Mn and Sr) were evaluated, and the element transport was calculated as a ratio between basolateral layer and bioaccessible contents, in percentage. The results are presented in Table 4.

Transport ranged from 0.8 to 3.2% and 1.2 to 3.1% for Mn and Sr, respectively. Soy hydrosoluble extract beverages presented the highest Mn and Sr bioavailability, and a similar behavior was observed in the bioaccessibility study (Figure 2). In general, low values (<3.2%) were found in this assay. It is well known that the Caco-2 cell model does not consider paracellular and passive transcellular transport, which are highly influenced by the organic species present in matrix [7]. Low transport was also reported for Ca in milk-based infant formulas [30] and Fe in soy-based yogurts [31]. Fe and Zn bioavailability were also evaluated in milk and soy-based infant formulas, ranging from 6.90 to 13.76% and 7.06 to 15.43% for Fe and Zn, respectively [32].

### 3.3. Phytic Acid in Soy-Based Beverages

For phytic acid determination, phosphorus content was extracted using HCl solution, and the determination was carried out by ICP OES. Inorganic phosphorus interference was minimized using an ionic exchange column [16,17,18]. Method accuracy was verified by recovery experiments using soy-based beverages with results ranging between 87 and 97%, in accordance with AOAC [23] and INMETRO [19] recommendations: 80–110%.

The mean phytic acid contents were: 2.09 mg/L, 7.33 mg/L, and 19.1 mg/L in isolate soy protein, soy hydrosoluble extract, and soybean beverages, respectively. The values were lower than those reported for soy-based beverages and soy sauce by Burgos-Luján and Tong [29], which were 0.935 and 5.35 g/L, respectively.

It is well known that trace element bioaccessibility and bioavailability are largely influenced by food components, such as carbohydrates, proteins, fibers, and phytates. Phytic acid may be found at 5% level, being one of the main storages of phosphorus in cereals, grains, and vegetables [33]. Al-Wahsh et al. [34] reported a correlation between oxalates (0.02 mg/g) and phytates (1.06 mg/g) and minerals (Ca + Mg) in their study with soy-based food (correlation coefficient = 0.90). In our study, a possible negative influence on Al bioaccessibility was found: the lowest values of phytic acid were found in soybeans and soy hydrosoluble extract samples, which presented the highest Al bioaccessible fractions.

### 3.4. Estimative of Risk Exposure to Inorganic Contaminants from Soy-Based Beverages Consumption

For the inorganic contaminants aluminum and strontium, the risk exposure assessment considered the daily intake of 200 mL of soy-based beverage and the FAO/WHO [35] recommend body weight (bw): adult = 60 kg and child = 15 kg and PTWI (Provisional Tolerable Weekly Intake) [36] for Al = 2 mg/kg bw, and the mean levels found in isolate soy protein beverages (Al = 0.76 mg/L, Sr = 0.36 mg/L), soy hydrosoluble extract beverages (Al = 0.61 mg/L, Sr = 0.14 mg/L), and soybean beverages (Al = 0.18 mg/L, Sr = 0.61 mg/L). The results are described in Table 5.

Overall, the estimation of Al and Sr exposure were found at safe levels. The highest Al values were found for isolate soy protein beverages, which can contribute to 3.5% and 0.9% PTWI for children and adults, respectively. For Sr, daily exposure from soybean beverages is up to 0.057 mg/kg bw for children and 0.014 mg/kg bw for adults. Although safe levels were found in this estimate of Al and Sr dietary exposure, it is important to highlight that several factors may also contribute to the contaminant exposure, such as nutritional condition, age, and meal composition [37].

### 3.5. Contribution for Minerals Daily Intake from Soy-Based Beverages Consumption

For copper, iron, manganese, selenium, and zinc, the contribution for minerals daily intake considered the daily intake of one glass of soy-based beverage (200 mL) and the mean levels found in isolate soy protein beverages (Cu = 0.11 mg/L, Fe = 0.87 mg/L, Mn = 0.22 mg/L, Se = 0.063 mg/L, Zn = 4.21 mg/L), soy hydrosoluble extract beverages (Cu = 0.20 mg/L, Fe = 10.8 mg/L, Mn = 0.50 mg/L, Se = 0.061 mg/L, Zn = 0.73 mg/L), and soybean beverages (Cu = 0.19 mg/L, Fe = 0.81 mg/L, Mn = 0.35 mg/L, Se = 0.055 mg/L, Zn = 5.03 mg/L). The values were compared to Brazilian [38] DRI (Dietary Reference Intake) for 4–8 years old children: Cu = 440 µg; Fe = 10 mg; Mn = 1.5 mg; Se = 30 µg; Zn = 5 mg, and for adults: Cu = 900 µg; Fe = 14 mg; Mn = 3 mg; Se = 60 µg; Zn = 11 mg. The results are shown in Table 6.

From Table 6, a daily intake of one glass (200 mL) of soy-based beverage may contribute up to 9% (Cu), 22% (Fe), 7% (Mn), 42% (Se), and 20% (Zn) of DRI for children, and up to 4% (Cu), 15% (Fe), 3% (Mn), 21% (Se), and 9% (Zn) of DRI for adults, according to the soy source. Overall, high contributions for DRI were observed in soy-based beverages, especially for Fe, Se, and Zn considering children’s consumption (17% and 20% for isolate soy protein and soybeans beverages, respectively). Thus, according to Brazilian regulation [38], soy-based beverages can be considered source of Fe, Se, and Zn (DRI > 15%) and represent a good alternative for the intake of these nutrients.

## 4. Conclusions

In this study, trace element (Al, As, Cd, Co, Cr, Cu, Fe, Li, Mn, Ni, Pb, Sb, Se, Sn, Sr, and Zn) occurrence and the effect of in vitro digestion in their bioaccessibility and bioavailability were investigated in soy-based beverages from Brazil. The trace elements profile indicated high levels of Al and Se in isolated soy protein beverages (1822 µg/L and 110 µg/L, respectively); Cu, Fe, and Mn in soy hydrosoluble extract beverages (268 µg/L, 11,385 µg/L and 665 µg/L, respectively), and Ni in soybean beverages (46 µg/L). Additionally, based on their total trace elements content, principal component analysis (PCA) classified soy-based beverages in three groups accordingly with their soy source (isolate protein, hydrosoluble extract, or beans).

The influence of in vitro digestion was evaluated by bioaccessibility (gastric and intestinal phases), bioavailability (Caco-2 cell culture model), and phytic acid antinutritional factor assays. Overall, Al, Cu, Fe, Mn, Se, Sr, and Zn bioaccessibility corresponded to approximately 40 to 80% of their total content. For Al and Se, bioaccessibility varied according to the soy source: isolate soy protein < soy hydrosoluble extract < soybeans. For Mn and Sr, bioavailability was higher in hydrosoluble extract beverages, corresponding up to 3.2% of the total content. Phytic acid results also indicated a possible negative influence on Al bioaccessibility.

For mineral dietary intake, soy-based beverages were found to be a source of Fe, Se, and Zn: values of dietary reference intake above 15% were estimated considering the daily consumption of one glass (200 mL). Nevertheless, the contribution for Al PTWI can reach 3.5% and 0.9% for children and adults, respectively. These results highlight the importance on performing bioaccessibility studies in order to provide a better understanding to the inorganic contaminants exposure and to the mineral intake from plant-based consumption.

## Figures and Tables

**Table 1:** Soy-based beverages—main ingredients.

Soy-Based Beverage	Brand ^1^	Main Ingredients
S1/S18	A	Water, sugar, concentrate grape juice, isolate soy protein, pectin, citric acid, vitamins, and zinc.
S2/S16	A	Water, sugar, concentrate apple juice, isolate soy protein, pectin, citric acid, vitamins, and zinc (S2)/calcium (S16).
S3/S17	A	Water, sugar, concentrate orange and peach juices, isolate soy protein, pectin, citric acid, vitamins, and zinc.
S4/S13	B	Water, hydrolysate soy extract, concentrate grape juice, sugar, vitamins, orange fiber, vitamins, iron, pectin, guar gum, and citric acid.
S5/S14	B	Water, hydrolysate soy extract, sugar, concentrate orange juice, vitamins, orange fiber, iron, pectin, guar gum, and citric acid.
S6/S15	B	Water, hydrolysate soy extract, sugar, apple pulp, citric acid, pectin, sucralose, iron, and vitamins.
S7/S10	C	Water, soybeans, invert sugar, concentrate orange juice, sugar, maltodextrin, vitamins, zinc, pectin, guar gum, citric acid, and sucralose.
S8/S11	C	Water, soybeans, invert sugar, concentrate apple juice, sugar, maltodextrin, vitamins, zinc, pectin, guar gum, citric acid, and sucralose.
S9/S12	C	Water, soybeans, invert sugar, concentrate grape juice, sugar, maltodextrin, vitamins, zinc, pectin, guar gum, citric acid, and sucralose.

^1^ Soy source: Brand A = Isolate protein; Brand B = Hydrolysate extract; Brand C = beans.

**Table 2:** ICP OES conditions for trace elements analyses.

Parameters	Conditions
Radiofrequency power (RF)	1.35 kW
Ar flow rate	12.0 L/min
Ar auxiliary flow rate	0.50 L/min
Nebulizer flow rate	0.55 L/min
Nebulizer	Seaspray
Spray chamber	Quartz double-pass
Wavelengths, nm (axial plasma view)	Sn, 189.925; As, 193.6906; Se, 196.026; Sb, 206.834; P, 213.618; Zn, 213.857; Cd, 214.439; Pb, 220.353; Ni, 221.648; Co, 228.615; Mn, 257.610; Fe, 259.940; Cr, 267.716; Cu, 324.754; Al, 396.152; Sr, 421.552; Li, 670.783

**Table 3:** Trace element contents in soy-based beverages.

Trace Element (µg/L)	Soy Isolate Protein (n = 6)	Soy Hydrosoluble Extract (n = 6)	Soybeans (n = 6)
Al	758 (137–1822) ^a^	609 (92–1375) ^a^	176 (45–356) ^a^
As	<38.2 ^a^	<38.2 ^a^	<38.2 ^a^
Cd	<3.8 ^a^	<3.8 ^a^	<3.8 ^a^
Co	1.6 (<9.0–9.8) ^a^	<9.0 ^a^	<9.0 ^a^
Cr	1.8 (<10.9–11.0) ^a^	<10.9 ^a^	<10.9 ^a^
Cu	109 (39–226) ^a^	195 (158–268) ^b^	194 (146–258) ^b^
Fe	871 (231–1473) ^a^	10,821 (9315–11,385) ^b^	814 (431–1254) ^a^
Li	<6.2 ^a^	<6.2 ^a^	<6.2 ^a^
Mn	216 (101–441) ^a^	495 (362–665) ^b^	346 (323–386) ^ab^
Ni	<25.7 ^a^	4.9 (<25.7–29.4) ^a^	29 (<25.7–46) ^b^
Pb	<10.9 ^a^	<10.9 ^a^	2.2 (<10.9–13) ^a^
Sb	6.2 (<10.3–24) ^a^	2.5 (<10.3–15) ^a^	12 (<10.3–61) ^a^
Se	63 (<26.7–110) ^a^	61 (36–93) ^a^	55 (37–72) ^a^
Sn	4.3 (<18.0–26) ^a^	<18.0 ^a^	<18.0 ^a^
Sr	359 (213–699) ^b^	142 (97–183) ^a^	606 (437–669) ^c^
Zn	4205 (226–5326) ^b^	735 (485–933) ^a^	5027 (4893–5208) ^ab^

^a, b, c^ Mean values between different columns with the same letter are not significantly different at *p* > 0.05, according to the Tukey’s test.

**Table 4:** Sr and Mn bioavailability using Caco-2 cells.

Soy Source	Analyte	Bioaccessible (µg/L)	Bioavailability	
			Basolateral (µg/L)	Transport (%)
Soybeans (n = 3)	Sr	306 ± 18	3.73 ± 1.62	1.2
	Mn	171 ± 8	2.01 ± 0.00	1.2
Hydrosoluble extract (n = 3)	Sr	33 ± 4	1.08 ± 0.25	3.1
	Mn	135 ± 11	4.20 ± 0.85	3.2
Isolate protein (n = 3)	Sr	163 ± 25	2.73 ± 0.08	1.7
	Mn	121 ± 34	0.97 ± 0.13	0.8

**Table 5:** Estimative of Al and Sr dietary exposure, considering a daily intake of one glass (200 mL) of soy-based beverages.

Soy Source	Weekly Consumption	Aluminum		Strontium	
		Children	Adults	Children	Adults
Isolate soy protein	mg/kg bw	0.071	0.018	0.033	0.008
	% PTWI	3.5	0.9	-	-
Hydrosoluble extract	mg/kg bw	0.057	0.014	0.013	0.003
	% PTWI	2.8	0.7	-	-
Soybeans	mg/kg bw	0.016	0.004	0.057	0.014
	% PTWI	0.8	0.2	-	-

PTWI (Provisional Tolerable Weekly Intake): Al = 2 mg/kg, bw (body weight), Sr = Not established [36].

**Table 6:** Cu, Fe, Mn, Se and Zn contribution to daily intake through consumption of one glass of soy-based beverages (200 mL).

Element	Isolate Soy Protein			Soy Hydrosoluble Extract			Soybeans		
	Intake (mg)	% DRI		Intake (mg)	% DRI		Intake (mg)	% DRI	
		Children	Adults		Children	Adults		Children	Adults
Cu	0.022	5	2	0.039	9	4	0.039	9	4
Fe	0.17	2	1	2.16	22	15	0.16	2	1
Mn	0.043	3	1	0.10	7	3	0.069	5	2
Se	0.013	42	21	0.012	41	20	0.011	37	18
Zn	0.84	17	8	0.15	3	1	1.01	20	9

DRI (Dietary Reference Intake [38]) for children: 4–8 years old (Cu = 440 µg; Fe = 10 mg; Mn = 1.5 mg; Se = 30 µg; Zn = 5 mg) and for adults (Cu = 900 µg; Fe = 14 mg; Mn = 3 mg; Se = 60 µg; Zn = 11 mg).

## Data Availability

The data that support the findings of this study are available upon reasonable request.
